# Evaluation of the effectiveness of surveillance policies to control the COVID-19 pandemic in São Paulo, Brazil

**DOI:** 10.1186/s41256-022-00260-4

**Published:** 2022-08-17

**Authors:** Lorena G. Barberia, Natália de P. Moreira, Brigina Kemp, Maria Amelia de Sousa Mascena Veras, Marcela Zamudio, Isabel Seelaender Costa Rosa, Rebeca de J. Carvalho, Tatiane C. M. Sousa

**Affiliations:** 1 Department of Political Science, University of São Paulo, Avenida Professor Luciano Gualberto, 315 - Sala 2067 - Cidade Universitária, São Paulo, SP 05508-900 Brazil; 2 Conselho de Secretários Municipais de Saúde - SP, Avenida Angélica, 2466, - 17° floor – Consolação, São Paulo, SP 01228200 Brazil; 3 Departamento de Saúde Coletiva, Faculdade de Ciências Médicas da Santa Casa de São Paulo, Dr Cesario Mota Jr St. 61, São Paulo, SP 01221-020 Brazil; 4 Department of Public Administration and Government, FGV EAESP Business Administration School of São Paulo, Avenida 9 de julho, 2029, Bela Vista, São Paulo, SP 01313-902 Brazil; 5 Fundação Oswaldo Cruz, ENSP, Rio de Janeiro, RJ Brazil

**Keywords:** COVID-19, Surveillance, RT-PCR public health testing policies

## Abstract

**Background:**

Surveillance efforts are essential to pandemic control, especially where the state is the primary health provider, such as Brazil. When public health testing guidelines limit molecular tests, there are reductions in detection efforts aimed at early recognition, isolation, and treatment of those infected with the virus. This study evaluates the effectiveness of surveillance policies to control the COVID-19 pandemic in São Paulo.

**Methods:**

We conducted an interrupted time series analysis with a segmented regression model to analyze if changes in the state’s guidelines improved RT-PCR testing outcomes in Brazil’s most affluent and largest state, São Paulo. Anonymized daily data on the RT-PCR tests conducted in public laboratories belonging to the state-wide network from March 1, 2020 to June 5, 2021 were extracted from the Sao Paulo State open-source database, while the data on the genomic sequences were obtained from GISAID. We then aggregated these data for the 17 regional health departments in the state to evaluate regional-level outcomes.

**Results:**

The public health system restricted RT-PCR testing to hospitalized cases in the first months. Testing was expanded to permit symptomatic testing of non-hospitalized persons only in July 2020, but a statistically significant increase in surveillance efforts was not observed. Case definition was expanded to allow case confirmation based on clinical, laboratory and image data criteria other than an RT-PCR test without increasing the testing effort for asymptomatic suspicious cases in September 2020. There was an increase in the mean volume of testing in each RHD, but the test positivity rate increased due to insufficient testing expansion. Results also show an uneven improvement in testing outcomes following these changes across the state’s regional health departments.

**Conclusions:**

Evidence suggests that lower RT-PCR testing and genomic surveillance efforts are associated with areas characterized by a higher population concentration and a greater population reliance on the public health system. Our results highlight the need to structure health surveillance and information systems for disease control and prevention in emergency settings considering local demographics and vulnerabilities. In high prevalence settings, efforts at identifying and including vulnerable populations in routine and enhanced surveillance programs during COVID-19 must be significantly improved.

## Introduction

Evidence from industrialized and low-resource countries has demonstrated that detection efforts to identify infected individuals are essential to an effective containment of COVID-19 along with policies aimed at movement restrictions that included border, school, and business closures, travel restrictions, restrictions on public gatherings, and local curfews [1–4]. There is growing consensus that coordinated and unified testing and genomic surveillance systems are needed to identify, track, and mitigate the transmission of SARS-CoV-2 and the variants of concern that have spread most rapidly. Molecular tests, commonly referred to as Reverse Transcription-Polymerase Chain Reaction (RT-PCR) tests, are considered the gold standard as they detect the virus’s RNA in infected individuals who are potentially transmitting the virus to other individuals [5]. RT-PCR and rapid lateral flow (antigen) tests are considered the first-best tests for diagnosis of COVID-19 and an essential part of a surveillance strategy that should include identifying and isolating infected individuals and ensuring the quarantine of close contacts. RT-PCR–positive samples are also the source used to conduct genomic surveillance, including the monitoring of variants of interest (VOI) and of concern (VOC) [6].

However, since the onset of the pandemic, the sizable volume of resources (monetary, laboratory, test kits) and continuous surveillance policies have been identified as significant impediments to the effective use of RT-PCR tests to contain the COVID-19 pandemic in poor and middle-income countries [7, 8]. As the developing world struggles to contain the spread of SARS-CoV-2 and faces an increasing number of fatalities [9], enhanced understanding is needed about whether specific diagnostic interventions are improving local epidemic control efforts.

Although Brazil has less than 3 percent of the world’s population and COVID-19 arrived relatively later than in Asia, Europe, and North America, the country has registered almost 10% of the world’s cases (over 21 million) and nearly 13% of all deaths (588,597) by July 2021 [10]. In this study, we analyze RT-PCR public health network testing data in the first 15 months of the COVID-19 pandemic in the state of São Paulo to assess surveillance efforts. São Paulo state, the most populous state in Brazil with over 40 million inhabitants, is the epicenter of the pandemic in the country [11], and 62 (%) of its population depends exclusively on the public health system [12]. To do so, we evaluate the effectiveness of surveillance policies to control the COVID-19 pandemic in São Paulo, considering the regional differences among the 17 Health Departments between March 2020 to June 2021.

## Methods

### Study setting

This study was conducted in São Paulo state, the most affluent and populated state in Brazil. The state of São Paulo has 20% of the Brazilian population but recorded 25% of deaths from COVID-19 in the country in two years of the pandemic, although it has the highest GDP and hospital coverage in the country [13, 14]. In addition to studying surveillance policies in the state of São Paulo, we also carried out a regional analysis covering the 17 Health Departments of the state from March 2020 to June 2021.

We analyzed the surveillance efforts provided by public health services since the Brazilian Health System is public and universal, known as *Sistema Único de Saúde (SUS)*. The management of the SUS is conducted by all government levels (federal, states, and municipalities) with different responsibilities. The surveillance and information health systems are under the responsibility of public managers, and considering the specificities of the state of São Paulo, it has the largest infrastructure of health services among all the other 26 units of the federation [15].

### Research design

We conducted a cross-sectional time-series study to evaluate the surveillance interventions effects between March 2020 to June 2021 across the 17 Health Departments of the São Paulo State [16]. This manuscript was based on the Strengthening the Reporting of Observational Studies in Epidemiology (STROBE) guidelines [17].

### Variables

To identify variables for RT-PCR testing outcomes, we analyzed WHO, ECDC, and CDC technical guidelines and daily press conferences that specifically addressed testing [18–23] and other academic publications and platforms [24–26]. Based on these documents, we identified a total of nine indicators (see Table 1). Of the nine indicators, two monitor variant surveillance as these efforts determine if emerging mutations render the virus more contagious, more potent, or resistant to existing vaccines and medicines—the remainder focus on testing outcomes and laboratory capacities at the regional level. We constructed a weekly time-series for each testing outcome indicator during each epidemiological week (Sunday-Saturday), the period for each of the seventeen Regional Health Departments (RHD) in the state. The RHD is the central territorial entity in which the public health system is organized in Brazil. In the State of Sao Paulo, the median number of municipalities in an RHD is 39 (minimum 9 and maximum 102).

**Table 1:** Surveillance efforts of São Paulo State (Brazil) from March 1, 2020, to June 5, 2021

	Overall	Testing policy I. March 17, June 30, 2020 Testing limited to health professionals and hospitalized cases [27]	Testing policy II July 1-September 14, 2020 (testing expanded to ambulatory symptomatic cases) [28]	Testing policy III September 15, 2020-February 25, 2021 (case definition expanded to permit case confirmation based on clinical, laboratory and image data criteria other than RT-PCR test) [29]	Testing policy IV February 26, 2021-June 5, 2021 (genomic surveillance guidelines were introduced) [30]
*RT-PCR tests per 100,000*^*a,b*^					
Weekly average	168.41	29.99	166.23	197.41	305.52
Standard deviation	134.27	31.18	92.50	97.59	123.66
*Weekly COVID-19 cases per 100,000*^*c*^					
Weekly average	129.95	29.82	141.86	126.93	260.69
Standard deviation	101.76	39.54	60.52	66.30	74.05
*RT-PCR tests/COVID-19 cases*^*c*^					
Weekly average	3.06	4.96	1.23	1.83	1.20
Standard deviation	14.97	16.27	0.54	1.34	0.43
*RT-PCR tests positivity rate (%)*					
Weekly average	31.21	25.67	32.21	31.22	41.79
Standard deviation	12.38	12.87	7.69	9.75	6.83
*No. of labs in RHDs*					
0	5				
> = 1	12				
Test Processing Time	No information available				
*Weekly Total RT-PCR Test Laboratory Processing Capacity (unit)*					
Weekly average	72,445.33	25,760	48,363.64	93,112.17	121,758
Standard deviation	39,317.76	13,236.52	10,129.08	8985.50	0
*No. of genomic sequences collected and uploaded*					
Weekly average	1.56	1.30	0.37	1.77	2.68
Standard deviation	4.49	4.94	1.24	4.92	4.92
*Average lag from collection to submissions to GISAID of SARS-CoV-2 genome by surveillance agencies (days)*					
Weekly average	89.05	180.28	157.12	64.16	55.59
Standard deviation	(61.20)	(48.35)	(41.16)	(38.28)	(20.97)

Data is aggregated by week

^a^No data were identified on the number of individuals whose RT-PCR tests were repeated, nor were data identified regarding the number of contacts tracked for each positive test result, or the number of tests processed by each laboratory. For this reason, these indicators have no score assigned

^b^The data are relative to the specimens collected. No data is available for testing encounters per individual reported by the State of São Paulo

^c^Covid-19 cases are the notified cases of the disease according to the database of the state health department

Based on a review of official state legislation, we identified four major testing interventions that were introduced by the State of São Paulo’s RT-PCR surveillance program: Testing limited to hospitalized cases and health professionals (Testing Policy I), which was published on March 17, 2020 [27]; Testing expanded to ambulatory symptomatic cases (Testing Policy II), which commenced on July 1, 2020 [28]; Inclusion of the clinical and epidemiological criteria as COVID-19 notification, in addition to RT-PCR testing (Testing Policy III) which was announced on September 15, 2020 [29]; and, genomic surveillance guidelines were introduced (Testing Policy IV), which were initiated on February 26, 2021 [30].

To identify if testing is associated with specific spatial and socioeconomic patterns in the public health networks across RHDs, we collected data on the 2020 population, and social vulnerability was provided from the Foundation for Statewide System Data Analysis [12]. The 2010 Social Vulnerability Index of São Paulo State is based on five socioeconomic and four demographic indicators. Social vulnerability is measured as the percentage of people living in each of the 17 RHD under high or very high social vulnerability (including urban and rural areas). For each RHD, these values represent the mean value of all municipalities in the specific region. We also obtained the proportion of the population which was exclusively dependent on the Brazilian public health system, known as *Sistema Único de Saúde* (SUS) in Portuguese, in May 2020. The per capita income estimates are based on 2010 census data.

### Data collection and processing

Anonymized daily data on the RT-PCR tests conducted in the public laboratories belonging to the state-wide network from March 1, 2020 to June 5, 2021 were extracted from the Sao Paulo State Health Secretariat’s Intelligent Monitoring System (SIMI) [31]. This open-source database provides the number of RT-PCR performed in the network of public labs for hospitalized and ambulatory patients and the overall confirmed daily case count. Data on the laboratory processing capacity of these labs were also collected from the same source. The data on the genomic sequences collected and deposited by the Instituto Adolfo Lutz network, which is responsible for sequencing the cases identified by the Center for Epidemiological Surveillance (CVE) for genomic surveillance in São Paulo state, were obtained from the Global Initiative on Sharing All Influenza Data (GISAID), which is the platform used for registering SARS-CoV-2 sequences by Brazilian institutes [32]. Since the objective of this study is to evaluate regional-level outcomes, the data were aggregated for the 17 regional health departments in the state.

### Data analysis

**Fig. 1:**
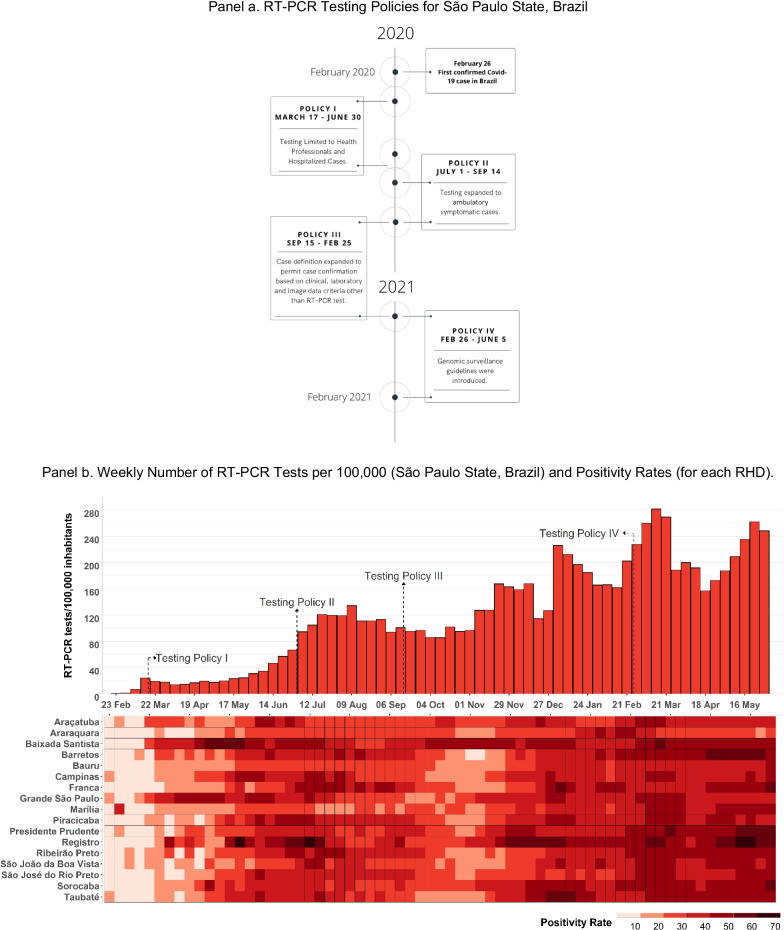

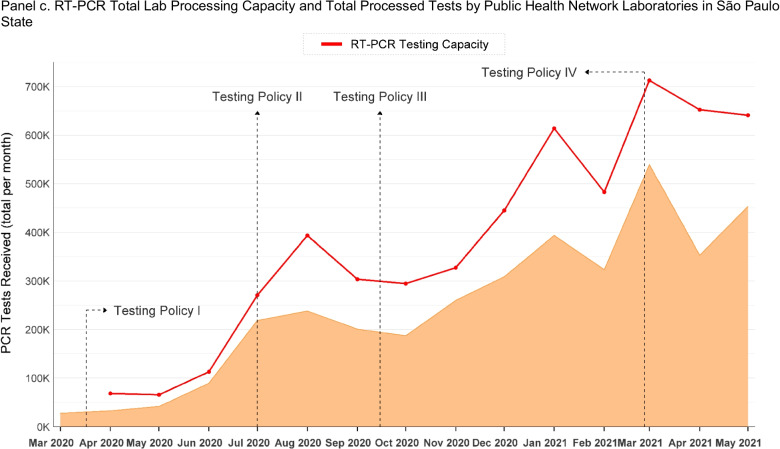
RT-PCR Testing Policies, Tests, Test Positivity, and Laboratory Testing Capacity in the Public Health Network of São Paulo State, Brazil and in each RHD (March 1, 2020–June 5, 2021)

We reconstructed time series data to estimate the mean change in testing indicators and 95(%) confidence intervals (95% CIs) for the state, and for each of the 17 RHDs between March 1, 2020 and June 5, 2021. We then analyzed several RT-PCR testing effort variables in the period between the onset of each policy and the announcement of the revised policy, as the study’s objective was to analyze whether each intervention positively interfered in the increase in the diagnostic testing effort. Therefore, we analyzed the following periods: Testing Policy I: March 17—June 30, 2020; Testing Policy II: July 1—September 14, 2020; Testing Policy III: September 15, 2020—February 25, 2021; and, Testing Policy III: February 26—June 5, 2021(Fig. 1, Panel a). These policies were identified through searches in the archive of the official webpage of the State of São Paulo, using the search terms “RT-PCR,” “SARS-Cov-2,” “test,” “genomic surveillance,” and “diagnosis” for the period between March 1, 2020, until June 30, 2021.

To do so, we conducted an interrupted time series analysis with a segmented regression model [33] to analyze if testing guidelines (Testing Policy II and III) produced changes in testing outcomes in each RHD and for the state overall. In other words, the same model was fitted to data from each RHD and for the entire state. We estimated the level and trend of the reported outcome testing indicator following testing policy interventions in 2020 for the state overall and for each RHD. An interrupted time series analysis of genomic surveillance (Testing Policy IV) in 2021 was restricted to the state overall as the small sample sizes limit more robust granular statistical analyses to be undertaken for this specific outcome. The analyses and figures were conducted using Stata version 16.1 and R 4.1.1.

## Results

### Surveillance policy efforts over time

The first confirmed case in Sao Paulo state was February 26, 2020. International importations accounted for a majority of cases at the outbreak’s start before local cases were detected [34]. Following the outbreak’s spread to local community transmission, there were four major policy changes in the state’s RT-PCR testing guidelines. From March 17 to June 30, 2020, state surveillance policies limited RT-PCR use to hospitalized and symptomatic individuals and health professionals. Under Testing Policy I, an average of 30 tests per 100,000 were conducted, and an average positivity rate of 25.67(%) was observed (Table 1). When São Paulo state expanded RT-PCR testing to permit testing of non-hospitalized, symptomatic cases (Policy II), an average of 166.23 tests per 100,000 were performed, and the positivity rate of 32.21(%) was observed from July 1, 2020 to September 14, 2020. There was a significant increase in COVID-19 cases during this period, with a peak in the average cases registered per 100,000 in weeks 32 and 33 of 2020. On September 15, 2020, the Brazilian Ministry of Health expanded the notification criteria, including clinical and epidemiological criteria (Policy III). This could have resulted in an expansion of the testing effort for asymptomatic and suspicious cases. However, an average of 197.41 tests per 100, 000 between September 15, 2020 to February 25 2021 were registered. In this period, the average positivity rate of 31.22(%) ranged from a weekly average rate of 19.76(%) (week 45, 2020) to 40.58(%) (week one, 2021) (Fig. 1, Panel b). The second highest peak in cases was observed in weeks two and four of 2021. When guidelines were revised to incorporate genomic surveillance as part of the state’s RT-PCR testing strategy from February 26 to June 5, 2021 (Policy IV), an average of 305.52 tests per 100,000 were conducted, and an average positivity rate of 41.80(%) was observed. This period coincides with the third highest peak in cases observed in week 22 of 2021.

### Regional analyses

Significant variation in the volume of tests conducted across the state’s 17 Regional Health Departments (RHD) over the past fifteen months is observed. From March 1, 2020 to June 5, 2021, a weekly average of 168.41 RT-PCR tests per 100,000 were performed across the state. São José do Rio Preto was the RHD with the highest average of tests performed (322.07 tests per 100,000), while Grande São Paulo was the lowest average of tests conducted (74.18 tests per 100,000). There has also been a substantial variation in the positivity rate across the regions. The state average positivity rate was 31.21(%) throughout the period. Araraquara presented the lowest average positivity rate (23.16%) and Registro the highest average positivity rate (40.55%).

In the early months of the pandemic, public health testing efforts were coordinated by the Instituto Adolfo Lutz. In April 2020, a new public laboratory system was created with the Instituto Butantan as its central manager [35] to complement efforts undertaken in the IAL network. In this period, university laboratories and two private labs were incorporated into the public laboratory network to expand testing. Notwithstanding the expansion of the network and central management, public laboratories remain unequally distributed across the São Paulo state territory. Only 12 of the state’s 17 Regional Health Departments (RHD) have at least one local laboratory in their region. While Grande São Paulo has four local laboratories (Instituto Adolfo Lutz Central, Instituto Adolfo Lutz Santo André, Instituto Biológico, and Instituto Butantan), five RHD do not have a local laboratory (Barretos, Franca, Registro, São João da Boa Vista, and Taubaté). There was considerable delay in expanding public health laboratory capacity in the state. Initially, RT-PCR tests were limited to processing 1,200 tests per day in the Instituto Adolfo Lutz (IAL) lab (Fig. 1, Panel c). By September 2020, the public laboratory network had expanded its processing capacity to 13,154 RT-PCR tests per day. In February 2021, the state reached a capacity to process 17,394 tests per day. No further increases were observed after this date.

The state’s public laboratories processing capacity is also unevenly distributed. Some laboratories have still not acquired the capacity to process tests (Instituto Adolfo Lutz Araçatuba and Instituto Adolfo Lutz Campinas). The two public laboratories with the highest daily processing capacity (Instituto Adolfo Lutz Central with a capacity of 1,100 tests and the Instituto Butantan with 4,500 tests) are in the same RHD (Grande São Paulo). The State of São Paulo also contracts private laboratories to process RT-PCR tests. These providers (DASA and Grupo Fleury) were contracted to process 1,945 tests per day in 2020. In 2021, private laboratories were contracted to process 3,545 tests per day (20.1% of daily testing in the network). In contrast, there was a smaller increase in the IAL network testing capacity (2,304 tests per day in September 2020 to 2,895 tests per day by February 2021). Data are only published on the date and the lab that received the RT-PCR test. The lab processing and test result date are not informed in public data sources.

Between March 1, 2020, and June 5, 2021, 1,763 samples were collected for genomic sequencing from RT-PCR positive samples by the Instituto Adolfo Lutz network in the state of São Paulo (18.06% of the 9 761 samples collected and 20.65% of the 6,324 genomes sequenced in this period in GISAID for São Paulo state). However, given delays in uploading sequences to the registry database and missing values, only 1,299 of these genomic sequences were deposited with GISAID in the same period. On average, 0.0024 of RT-PCR positive tests in each RHD in the state of São Paulo were sequenced per week. In 2020, the number of sequences registered in GISAID was limited. On average, 0.70 sequences per RHD were registered per week in 2020 (based on 0.0025 of RT-PCR positive tests). While, on average, 3.32 sequences were registered per week in 2021 (based on 0.0023 of RT-PCR positive tests). The highest weekly average was observed in week six, 2021, when 9.12 genomic sequences were performed across the RHDs. Most of the sequences were uploaded to GISAID with considerable delay. The time lag between the specimen collection and submission has been remarkably high in 2020 (on average, 149.3 days). Since March 2021, there has been a marked rise in sequencing and submission. The time lag has substantially decreased in 2021 (54.4 days from specimen collection to submission).

**Fig. 2:**
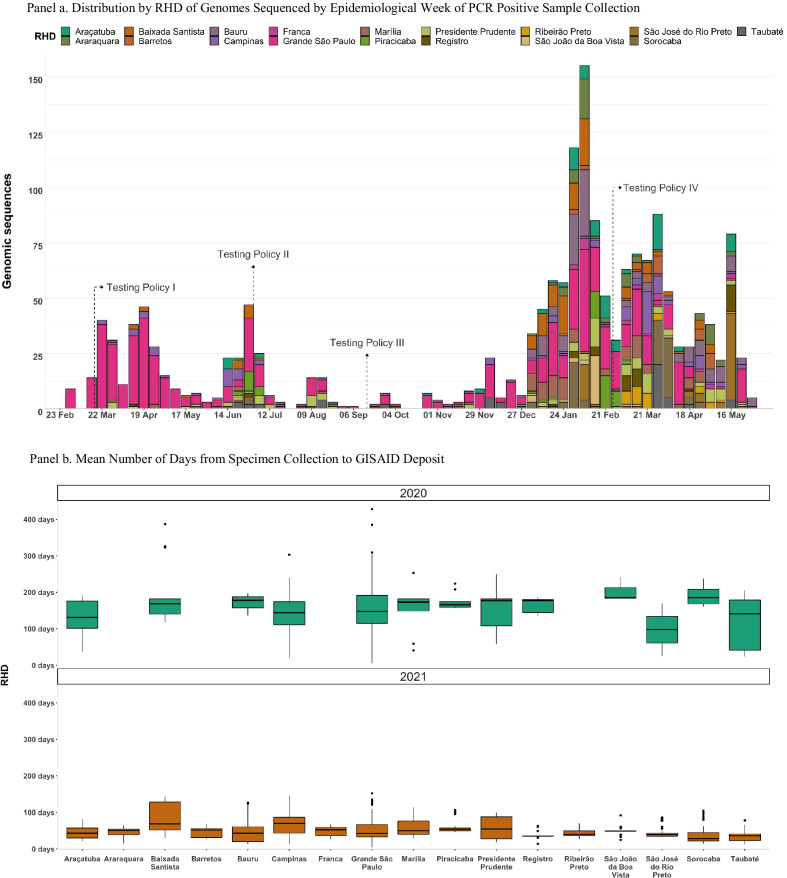
Genomic Sequencing in São Paulo State, Brazil (March 1, 2020–June 5, 2021)

Genomic sequencing by the IAL varies considerably across the state’s RHDs (Fig. 2, panel a). Grande São Paulo was the RHD with the highest weekly average of genomic sequencing performed (on average, 9.54 per week), while Franca was the lowest average of genomic sequencing conducted (on average, 0.33 per week). The lag in time from collection to submissions of the SARS-CoV-2 genome to GISAID by the IAL network has also varied significantly across RHDs (Fig. 2, Panel b). In 2020, the lowest average weekly time was 97.50 days from samples in São José do Rio Preto, and the highest average was 210 days from samples collected from Baixada Santista. In 2021, this period was considerably reduced. The IAL network performed the lowest average weekly time in Registro (41 days) and the highest average in Baixada Santista (68 days). The available data do not permit verification of whether the difference in processing time is due to which network laboratory is performing sequencing.

### Quantitative results

**Fig. 3:**
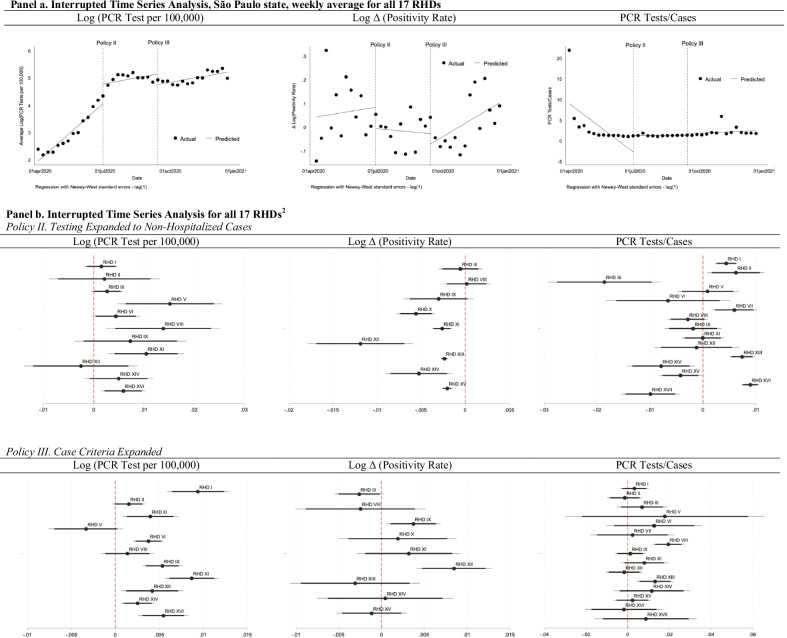
Segmented Regression for the RT-PCR Testing Policies in the Public Health Network. *Notes* Data from Public Health Network of São Paulo State, Brazil and in each RHD from March 29, 2020, to December 26, 2020. The figure reports the coefficients and 95% and 90% confidence interval of the post intervention linear trend estimated via interrupted time series analysis for Policy II (Testing Expanded to Non-Hospitalized Cases) and Policy III (Case Criteria Expanded). We excluded from the analysis the RHDs in which serial correlation was detected using the Cumby-Huzinga test. RHD I (Grande São Paulo), II (Araçatuba), III (Araraquara), IV (Baixada Santista), V (Barretos), VI (Bauru), VII (Campinas), VIII (Franca), IX (Marília), X (Piracicaba), XI (Presidente Prudente), XII (Registro), XIII (Ribeirão Preto), XIV (São João da Boa Vista), XV (São José do Rio Preto), XVI (Sorocaba), and XVII (Taubaté)

For the state overall, we transformed one of these dependent variables (RT-PCR tests Positivity Rate) into first differences of their natural logarithm, which is the per-week growth rate (Table 2). The results showed a 0.005 [95(%) confidence interval (CI) -0.003; 0.013] increase in the log of RT-PCR tests per 100,000, -0.0008 [95% confidence interval (CI) -0.0058; 0.0042] decrease in the ratio of RT-PCR tests per case, and a -0.0003(%) [95(%) confidence interval (CI) -0.0016; 0.001] decrease in the growth rate of the RT-PCR test positivity rate following Testing Policy II (Fig. 3, Panel a). In turn, Testing Policy III, results showed a 0.005 [95(%) confidence interval (CI) 0.002; 0.007] increase in the log of RT-PCR tests per 100,000, a 0.0076 [95(%) confidence interval (CI) -0.0046; 0.0197] increase in the ratio of RT-PCR tests per case, and an 0.002(%) [95(%) confidence interval (CI) 0.0004; 0.003(%)] increase in the growth rate of the RT-PCR positivity rate. As a result, Testing Policy II did not produce a statistically significant increase in surveillance efforts. Following Testing Policy III, there was an increase in the mean volume of testing in each RHD, but the test positivity rate increased due to insufficient testing expansion.

**Table 2:** Estimated Coefficients for the Segmented Regressions for RT-PCR Testing Policies in São Paulo (Brazil)

Dependent variables of RT-PCR testing outcome:	I. Log (RT-PCR tests per 100,000)		II.$\frac{RT - PCR Tests}{Cases}$		III. Log (positivity rate)	
	Coefficient	Newey standard error	Coefficient	Newey standard error	Coefficient	Newey standard error
Intercept	1.948	(0.158)***	8.961	(3.988)**	0.043	(0.090)
Time	0.023	(0.003)***	− 0.129	(0.069)*	0.000	(0.002)
Testing policy II	0.710	(0.278)**	4.018	(2.576)	− 0.090	(0.081)
Time*post testing policy II	− 0.018	(0.005)***	0.128	(0.069)*	− 0.001	(0.002)
Testing policy III	− 0.392	(0.188)**	0.481	(0.394)	− 0.045	(0.047)
Time*post testing policy II*post testing policy III	− 0.000	(0.004)	0.008	(0.006)	0.002	(0.001)**
*Post intervention linear trend*						
*Testing policy II (*β1 + β3*)*	0.005	(0.004)	− 0.001	(0.002)	− 0.000	(0.001)
*Testing policy III (*β1 + β3 + β5*)*	0.005	(0.001)**	0.007	(0.006)	0.002	(0.001)**
Number of observations	39		39		39	

From March 29, 2020 to December 26, 2020 (aggregated data by week)

For each dependent variable, the following segmented regression model was estimated: Y_t_ = β0 + β1 time + β2Testing Policy II_t_ + β3 time*post Testing Policy II_t_ + β4 Testing Policy III_t_ + β5 time*post Testing Policy IIIt + ε_t_

***p-value < 0.001, **p-value < 0.05, *p-value < 0.1

Uneven improvements in testing outcomes were observed across the state (Fig. 3, Panel b). Following Testing Policy II, an increase in the log of RT-PCR tests per 100,000 was only observed in four RHDs (RHD V, VIII, XI, and XVI) and a reduction in the log of RT-PCR tests positivity rate occurred in six RHDs (X, XI, XII, XIII, XIV, and XV). With respect to the ratio of RT-PCR tests per case, a decrease was observed in four RHDs (III, XIV, XV, and XVII) and an increase in five RHDs (I, II, VII, XIII, and XVI) after Testing Policy II. Under Testing Policy III, there was an increase in the log of RT-PCR tests per 100,000 in eight RHDs (I, III, VI, IX, XI, XII, XIV, XVI), and no reductions in test positivity rates were observed. Instead, test positivity rates increased after Testing Policy III in two RHDs (IX, XII). After Testing Policy III, the ratio of RT-PCR tests per case increased in two RHDs (VIII and XIII). These results are only with respect to those RHDs in which no evidence of first or second-order autocorrelation was found in the residuals after using Newey-Standard Errors for first-order serial correlation.

The impact of genomic surveillance efforts was analyzed based on the number of samples and the time delay between sample collection and deposit to the GISAID (Table 3). Testing Policy IV did not produce a statistically significant increase in genomic surveillance efforts. Results showed a -0.020 [95(%) confidence interval (CI) -0.046; 0.005] decrease in the number of samples and a 0.028 [95(%) confidence interval (CI) -0.142; 0.198] increase in the time delay between sample collection and deposit to the GISAID platform after the introduction of the policy.

**Table 3:** Estimated Coefficients for the Segmented Regression for Genomic Surveillance Policies in São Paulo State (Brazil)^1^

Dependent Variables of RT-PCR Testing Outcome:	I. Number of samples		II. Time delay between sample collection and deposit to the GISAID	
	Coefficient	Newey Standard Error	Coefficient	Newey Standard Error
Intercept	0.730	(0.443)	56.687	(2.481)***
Time	0.138	(0.033)**	− 0.155	(0.141)
Testing Policy IV	− 4.731	(1.582)**	7.509	(6.794)
Time*Post Testing Policy IV	− 0.158	(0.037)***	0.184	(0.170)
*Post intervention linear trend*				
*Testing Policy IV* *(*β1 + β3*)*	− 0.020	(0.012)	0.028	(0.081)
Number of Epidemiological Weeks		23		23

From December 27, 2020 to July 05, 2021^1^ (aggregated data by week)

For each dependent variable, the following segmented regression model was estimated: Y_t_ = β0 + β1 time + β2Testing Policy IV_t_ + β3 time*post Testing Policy IV_t_ + ε_t_

***p-value < 0.001, **p-value < 0.05, *p-value < 0.1

Considering the results related to the four analyzed policies, there is no significant association between the public RT-PCR testing variables and each policy. Figure 1 also shows no improvement in the testing effort in the period studied, even though the previous policies were maintained and could have shown accumulated positive effects, with an improvement in all RT-PCR testing effort variables.

**Fig. 4:**
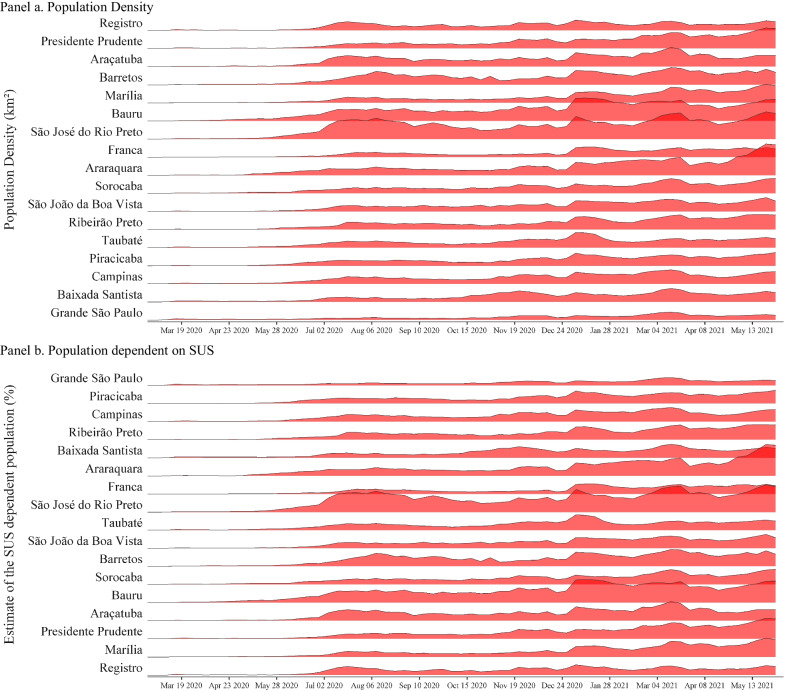

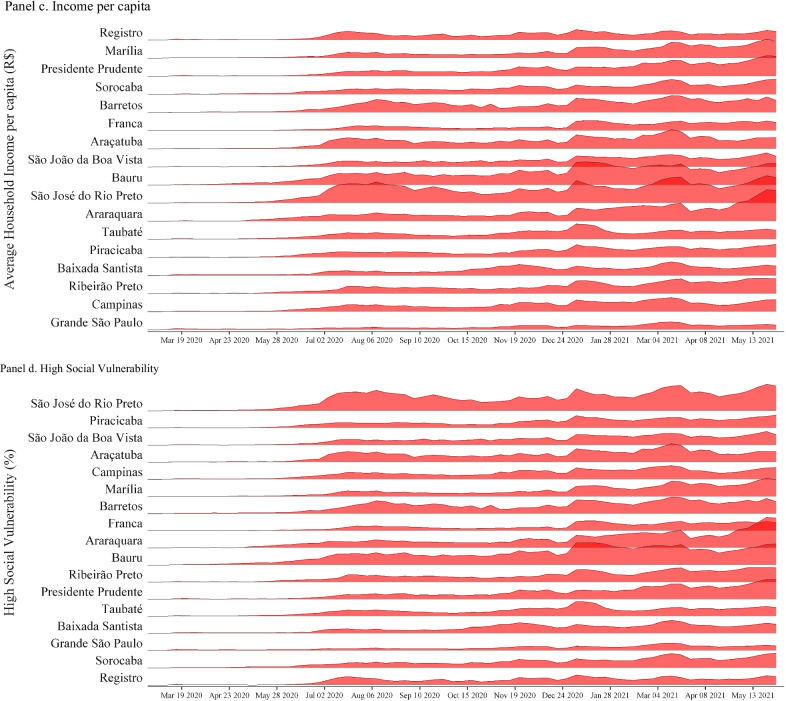
Variation in Testing per 100,000 and Sociodemographic Indicators across Epidemiological Weeks in the Regional Health Departments. *Notes* Data from RHDs of São Paulo State, Brazil, from March 1, 2020 to June 5, 2021. RHDS ranked from lowest to highest in each panel

RT-PCR tests per 100,000 are negatively correlated with high socioeconomic vulnerability (Pearson correlation coefficient = − 0.184, *p* < 0.001) and with a higher population density (Pearson correlation coefficient = − 0.196, *p* < 0.001). RT-PCR test positivity rate is positively correlated with high socioeconomic vulnerability (Pearson correlation coefficient = 0.251, *p* < 0.001) and with a higher population density (Pearson correlation coefficient = 0.0744, *p*-value = 0.013), and it is negatively correlated with average income per capita (Pearson correlation coefficient = − 0.066, *p*-value = 0.026) (see Fig. 4). COVID-19 cases, in turn, are negatively correlated with high socioeconomic vulnerability (Pearson correlation coefficient = − 0.066, *p*-value = 0.027) and higher population density (Pearson correlation coefficient = − 0.099, *p* < 0.001). The number of genomic sequences collected and uploaded at the GISAID is positively correlated with high socioeconomic vulnerability (Pearson correlation coefficient = 0.067, *p*-value = 0.025), higher population density (Pearson correlation coefficient = 0.437, *p* < 0.001), and income per capita (Pearson correlation coefficient = 0.263,* p* < 0.001), and it is negatively associated with a higher population dependent on SUS (Pearson correlation coefficient = − 0.143, *p* < 0.001). The average time delay between sample collection and deposit to the GISAID is positively correlated with high socioeconomic vulnerability (Pearson correlation coefficient = 0.136, *p*-value = 0.014), population density (Pearson correlation coefficient = 0.226, *p* < 0.001), and average income per capita (Pearson correlation coefficient = 0.167, *p*-value = 0.002).

## Discussion

When public health testing guidelines limit RT-PCRs, there are reductions in detection efforts aimed at early recognition, isolation, and treatment of those infected with SARS-CoV-2. In periods in which testing guidelines are expanded to authorize testing of a larger pool of suspected cases, we hypothesized that testing indicators would be associated with improvements in the incidence of COVID-19 infection. Findings across the state’s 17 regional health departments did not support our hypotheses based on the total number of RT-PCR tests conducted per 100,000, the test positivity rate, test processing time, proximity to local public health labs, as well as indicators to monitor surveillance efforts, including policies to conduct contact tracing, report testing outcomes based on repeated testing of individuals, and expand genomic surveillance.

There was limited improvement in surveillance efforts in the State of São Paulo. RT-PCR testing as a surveillance strategy was not strengthened as cases increased. A full month after the detection of the first case in the state, guidelines were issued to restrict testing to hospitalized patients with pneumonia and respiratory infection in late March 2020. The state expanded testing guidelines to include patients with respiratory infection evaluated in primary care settings in July 2020, but only after the first peak had occurred. Although test positivity rates remained above recommended maximum thresholds in subsequent weeks, the testing volume expanded gradually and sporadically. In most epidemiological weeks, testing remained below the public laboratory network’s processing capacity. Before the onset of the state’s second and most severe wave of cases and deaths, the regulation regarding COVID-19 notification was improved in September 2020 with the inclusion of clinical and epidemiological criteria. In comparison to the rest of Brazil, the state of Sao Paulo made early efforts to promote genomic surveillance. However, although genomic sequencing increased across RHDs (see Appendix 1), the speed of detection is not correlated with enhanced and targeted testing to further limit VOCs spread in the state. The information is also being shared with a considerable delay with the global health community.

To some extent, the observed variability in surveillance reflects the heterogeneity in Brazil’s most populated and well-endowed state. The Grande São Paulo region, for example, comprises 39 municipalities and has experienced high mortality rates [36]. In addition, in the Greater São Paulo region, where the state capital is located, there is an enormous private health service, including private laboratories. Despite the widespread availability of public laboratories compared to the rest of the state (and other settings in Brazil), the volume of testing was relatively low compared to other regions of the state. On the other hand, some RHDs with smaller populations and public health laboratory access improved their testing efforts considerably.

Incidence is impacted by physical distancing policies, their enforcement, and compliance [11, 37]. In the case of Sao Paulo, the state government coordinated the rolling implementation of various restrictions to increase distancing (starting in March 2020) across all regional health departments. When the government authorized the loosening of restrictions in June 2020, and throughout the subsequent period analyzed in this study, RT-PCR testing outcomes were not used in these assessments. Differing levels of public engagement in seeking testing across geographic settings and across times may differentially affect both individual and aggregate demand for diagnosis and reported test outcomes.

Our findings may help to explain the persistent and relatively high magnitude of cases observed in the State of Sao Paulo since the onset of the pandemic in early 2020. During the entire study period, no guidelines were identified to incorporate rapid, lateral flow antigen tests [38, 39] as an additional diagnostic test to increase the capacity to identify infectious individuals and more rapidly inform them of their testing status, especially in RHDs with limited local laboratory capacity. Despite growing evidence of its effectiveness in control and containment, a contact tracing program was not introduced with specific guidelines, nor were efforts incorporated to track secondary attack rates in the state [40].

Our study offers several contributions to how Brazilian surveillance policies and global health efforts to control the COVID-19 pandemic and other emergent diseases could be further strengthened. First, policies and resources must be directed at identifying infected individuals during their infectious period regardless of the severity of their symptoms, and these programs must further contribute resources to trace and test contacts and isolate positive cases. Second, the unequal distribution of existing infrastructure can lead to inadequate surveillance of important geographic territories and communities during an emergency. For this reason, investments must be directed at strengthening public laboratory capacities to enable speedy diagnostic testing, and to reduce their uneven distribution considering demographic and other determinants of vulnerability. Open-source databases with high quality and transparency are essential for the monitoring and improvement of surveillance efforts. Our study reveals that these systems were not significantly improved over the initial 16 months of the onset of the pandemic.

Our study has some limitations. First, high-quality, population-based health administrative databases are not publicly accessible in the State of São Paulo. Several indicators could not be evaluated as either restricted or no information was provided in the public databases accessed by this study. Second, the sample selection criteria by which positive cases are selected for sequencing are not disclosed to differentiate those cases selected based on random selection or other epidemiological criteria. Testing performed by private laboratories contracted by municipalities is not reported in the SIMI platform, nor are data on lateral flow tests conducted by municipalities in the public health system.

## Conclusions

In this study, we used nine indicators to measure RT-PCR testing in the 17 Regional Health Departments (RHD) of São Paulo state, the epicenter of the COVID-19 pandemic in Brazil. These indicators are based on recommendations by the World Health Organization (WHO), the European Center for Disease Control (ECDC), and the Centers for Disease Control and Prevention (CDC). Overall, we find limited evidence that testing guidelines improved surveillance in Brazil’s largest state, especially in poorer and more populous RHDs.

## Figures and Tables

**Fig. 5:**
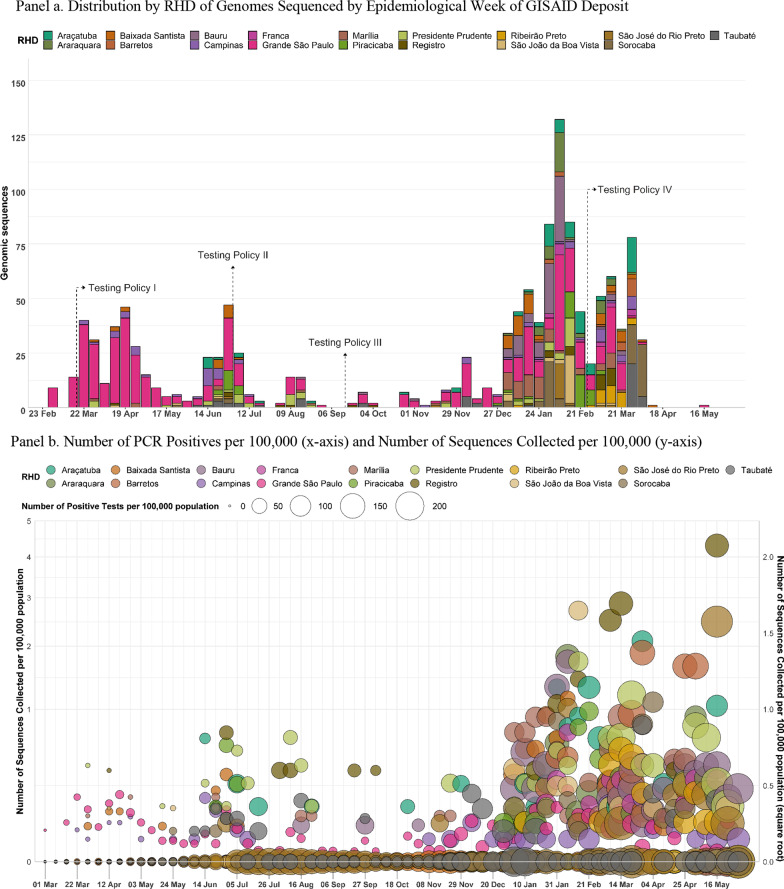

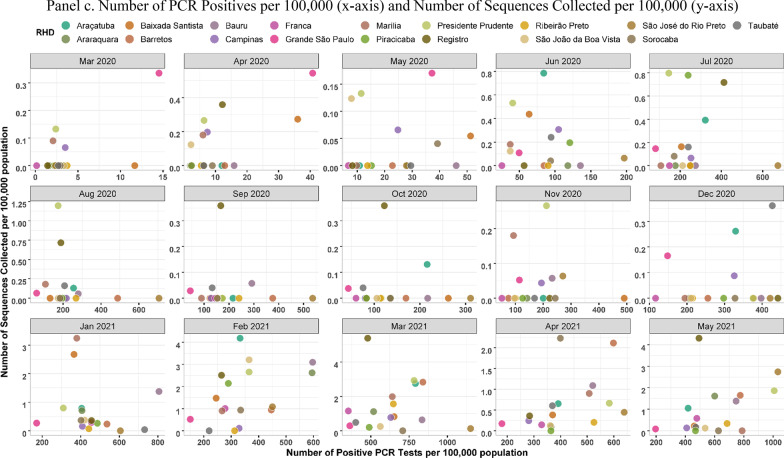
Genomic Sequencing in São Paulo State across Regional Health Departments and RT-PCR Test Positivity (March 1, 2020–June 5, 2021)

## Data Availability

The data and replication materials will be made available in the Harvard Dataverse.

## Appendix

See Fig. 5.

## References

[1] Kim S, Castro MC (2020). Spatiotemporal pattern of COVID-19 and government response in South Korea (as of May 31, 2020). Int J Infect Dis.

[2] Summers J, Cheng H-Y, Lin H-H, Barnard LT, Kvalsvig A, Wilson N, Baker MG (2020). Potential lessons from the Taiwan and New Zealand health responses to the COVID-19 pandemic. Lancet Reg Health West Pac.

[3] Crozier A, Rajan S, Buchan I, McKee M (2021). Put to the test: use of rapid testing technologies for covid-19. BMJ.

[4] Pitzer VE, Chitwood M, Havumaki J (2021). The impact of changes in diagnostic testing practices on estimates of COVID-19 transmission in the United States. Am J Epidemiol.

[5] Vandenberg O, Martiny D, Rochas O, van Belkum A, Kozlakidis Z (2021). Considerations for diagnostic COVID-19 tests. Nat Rev Microbiol.

[6] Novelli G, Biancolella M, Mehrian-Shai R (2021). COVID-19 one year into the pandemic: from genetics and genomics to therapy, vaccination, and policy. Hum Genom.

[7] Giri AK, Rana DR (2020). Charting the challenges behind the testing of COVID-19 in developing countries: Nepal as a case study. Biosaf Health.

[8] Werneck GL, Porto LC, Sena A, da Costa O, Junior F, Cavalcanti AC, Santos ÂMG, Secco DA, Silva M, Mariani D, Chieppe A, Tanuri A (2021). The incidence and geographical spread of SARS-CoV-2 in Rio de Janeiro, Brazil based on RT-PCR test results. Rev Soc Bras Med Trop.

[9] Ranzani OT, Bastos LS, Gelli JGM (2021). Characterisation of the first 250 000 hospital admissions for COVID-19 in Brazil: a retrospective analysis of nationwide data. Lancet Respir Med.

[10] Johns Hopkins University. Coronavirus Resource Center. 2021.

[11] Castro MC, Kim S, Barberia L (2021). Spatiotemporal pattern of COVID-19 spread in Brazil. Science.

[12] Fundação Estadual Sistema Estadual de Análise de Dados (SEADE). Data from: Sistema de projeções populacionais para os municípios do estado de São Paulo. 2020.

[13] IBGE (Brazilian Institute of Geography and Statistical). National accounts. Available at: https://www.ibge.gov.br/en/statistics/economic/national-accounts.html. (Last accessed 3 Feb 2022).

[14] Brazil. Ministry of Health. DATASUS. Available at: https://datasus.saude.gov.br/. (Last accessed 3 Feb 2022).

[15] Brazil. Lei 8080 de 19 de setembro de 1990. Available at: http://www.planalto.gov.br/ccivil_03/leis/l8080.htm. (Last accessed 3 Feb 2022).

[16] Wayne F, Roderick PM (1991). Cross-sectional time series designs: a general transformation approach. Multivar Behav Res.

[17] von Elm E, Altman DG, Egger M, Pocock SJ, Gøtzsche PC, Vandenbroucke JP (2008). The strengthening the reporting of observational studies in epidemiology (STROBE) statement: guidelines for reporting observational studies. J Clin Epidemiol.

[18] Centers for Disease Control and Prevention (CDC). SARS-CoV-2 testing strategy: considerations for non-healthcare workplaces. 2020. https://www.cdc.gov/coronavirus/2019-ncov/community/organizations/testing-non-healthcare-workplaces.html. (Last accessed 13 Sep 2021).

[19] Centers for Disease Control and Prevention (CDC). Evaluating case investigation and contact tracing success. 2020. https://www.cdc.gov/coronavirus/2019-ncov/php/contact-tracing/contact-tracing-plan/evaluating-success.html. (Last accessed 13 Sep 2021).

[20] European Center for Disease Prevention and Control (ECDC). Guidance for representative and targeted genomic SARS-CoV-2 monitoring. 2021. https://www.ecdc.europa.eu/en/news-events/ecdc-releases-new-dashboard-sars-cov-2-variants. (Last accessed 13 Sep 2021)

[21] World Health Organization (WHO). Public health criteria to adjust public health and social measures in the context of COVID-19 (Version from 12 May 2020). 2020.

[22] World Health Organization (WHO). Considerations for implementing and adjusting public health and social measures in the context of COVID-19 (Version 14 June 2021). 2021.

[23] Pan American Health Organization (PAHO). Diretrizes laboratoriais para detecção e diagnóstico de infecção pelo vírus da COVID-19. 2020. https://iris.paho.org/bitstream/handle/10665.2/52523/OPASIMSPHECOVID19200038_por.pdf?sequence=1&isAllowed=y (Last accessed 31 Aug 2021)

[24] Leal FE, Mendes-Correa MC, Buss LF (2021). Clinical features and natural history of the first 2073 suspected COVID-19 cases in the Corona São Caetano primary care programme: a prospective cohort study. BMJ Open.

[25] Harvard Global Health Institute (HGHI). Pandemics explained: unlocking evidence for better decision making. 2020. https://globalepidemics.org/key-metrics-for-covid-suppression/ (Last accessed 13 Sep 2021)

[26] Fiocruz-Instituto de Comunicação e Informação Científica e Tecnológica em Saúde (ICICT). MonitoraCovid-19. https://bigdata-covid19.icict.fiocruz.br/ (Last accessed 31 Aug 2021)

[27] São Paulo State Government. Resolução SS - 28, de 17-3-2020, published on March 17, 2020. Available at https://www.saopaulo.sp.gov.br/wp-content/uploads/2020/03/E_R-SS-CGOF-28_170320-1.pdf (February 9, 2022).

[28] São Paulo State Government. Deliberação CIB - 55, de 1º-7–2020, published on July 1, 2020. Available at http://saude.piracicaba.sp.gov.br/wp-content/uploads/2020/04/Delibera%C3%A7%C3%A3o-CIB-n%C2%BA.-552020.pdf. (9 Feb 2022).

[29] Brazil, Ministry of Health. Guia de Vigilância Epidemiológica – Emergência de Saúde Pública de Importância Nacional pela Doença pelo Coronavírus 2019, published on 5 Aug 2020

[30] Brazil, Ministry of Health. Vigilância genômica do vírus SARS-CoV-2 no âmbito da SVS/MS, published on February 20, 2021. Available at https://www.gov.br/saude/pt-br/coronavirus/publicacoes-tecnicas/guias-e-planos/vigilancia-genomica-do-virus-sars-cov-2. (9 Feb 2022).

[31] Governo de São Paulo. Data from: Sistema de Monitoramento Inteligente - SIMI. 2021.

[32] Global Initiative on Sharing All Influenza Data (GISAID). Data from: Genomic epidemiology of hCoV-19. 2021.

[33] Linden A (2015). Conducting interrupted time-series analysis for single-and multiple-group comparisons. Stand Genom Sci.

[34] Candido DS, Claro IM, De Jesus JG (2020). Evolution and epidemic spread of SARS-CoV-2 in Brazil. Science.

[35] Instituto Butantan. Governo de São Paulo lança plataforma de laboratórios para diagnóstico de Covid-19. 2020;

[36] Ribeiro KB, Ribeiro AF, de Sousa Mascena Veras MA, de Castro MC (2021). Social inequalities and COVID-19 mortality in the city of São Paulo, Brazil. Int J Epidemiol.

[37] Barberia LG, Cantarelli LGR, de Faria Oliveira MLC, de Paula Moreira N, Rosa ISC (2021). The Effect of state-level social distancing policy stringency on mobility in the states of Brazil. Revista de Administração Pública.

[38] Mina MJ, Andersen KG (2021). COVID-19 testing: one size does not fit all. Science.

[39] Mina MJ, Peto TE, García-Fiñana M, Semple MG, Buchan IE (2021). Clarifying the evidence on SARS-CoV-2 antigen rapid tests in public health responses to COVID-19. Lancet.

[40] Hellewell J, Abbott S, Gimma A (2020). Feasibility of controlling COVID-19 outbreaks by isolation of cases and contacts. Lancet Glob Health.

